# Inhibitors Alter the Stochasticity of Regulatory Proteins to Force Cells to Switch to the Other State in the Bistable System

**DOI:** 10.1038/s41598-017-04596-7

**Published:** 2017-06-30

**Authors:** Wun-Sin Jhang, Shih-Chiang Lo, Chen-Chao Yeh, Che-Chi Shu

**Affiliations:** Department of Chemical Engineering and Biotechnology, National Taipei University of Technology, Taipei, Taiwan

## Abstract

The cellular behaviors under the control of genetic circuits are subject to stochastic fluctuations, or noise. The stochasticity in gene regulation, far from a nuisance, has been gradually appreciated for its unusual function in cellular activities. In this work, with Chemical Master Equation (CME), we discovered that the addition of inhibitors altered the stochasticity of regulatory proteins. For a bistable system of a mutually inhibitory network, such a change of noise led to the migration of cells in the bimodal distribution. We proposed that the consumption of regulatory protein caused by the addition of inhibitor is not the only reason for pushing cells to the specific state; the change of the intracellular stochasticity is also the main cause for the redistribution. For the level of the inhibitor capable of driving 99% of cells, if there is no consumption of regulatory protein, 88% of cells were guided to the specific state. It implied that cells were pushed, by the inhibitor, to the specific state due to the change of stochasticity.

## Introduction

The important role of cell-to-cell variation in gene expression has received considerable attention in recent decades. Phenotypic population heterogeneity and the difference of cell fates mostly result from stochastic gene expression. Such noise-driven processes have a profound and lasting effect on networks of gene regulation. One of the influential consequences of internal noise is the bimodal distribution for system featured with bistability. The appearance of bimodal distributions indicates two different sub-populations (modes), one around the low end and the other the high end. A bistable system, the presence of two stable steady states within the certain range of parameter values, has been extensively studied because it arises in a wide range of biological systems^1–3^.

The system of bistability has binary response between ON and OFF stable steady states where ON often refers to high expression level and OFF to low level of gene expression. Consequently, the bimodal distribution appears in population as a result of the genetic toggle switch^3–5^ and its two modes reflects two stable steady states, the ON and the OFF states. A gene regulation established from two promoters in a mutually inhibitory network is the most popular motif of bistable systems^6–13^. It is composed of two inducible promoters where one is repressed by the protein from the other gene. With the presence of the repressor, the state of the promoter is inactive. Briefly, the high expression of one gene forces the other gene to be silent. This basic framework plays a critical role in various biological systems, including glucose consumption, viral infection and the decision of life cycle. The similar regulatory networks are employed by bacteria, yeast, or mammalian cells^1, 14–23^.

One major pattern of transcriptional regulation is the interaction between regulatory proteins and DNA. It is usually involving inhibitors acting on the regulatory proteins^24, 25^. A common scenario is that the binding of inhibitors causes the configuration change of the regulatory protein and alters the gene expression. However, the role of inhibitor does not merely consume the regulatory proteins. Interestingly, with the same averaged particle number of free reactant, the distribution is changed when other agents are competing for the reactant^26^. Without a doubt, while the inhibitors compete for the regulatory protein, the stochastic fluctuations of protein are changed.

Stochastic fluctuations are gradually appreciated in recent years for its indispensable role in many cellular activities^24, 27^ and it is vital for the bimodal distribution. For bistable systems, the noise-induced shifts of states are the main causes of a bimodal distribution. Moreover, the increase of stochastic fluctuations leads to the decrease of the mean first passage time^28, 29^. Recently, instead of altering the concentration of the critical intracellular variable, it has been reported that the manipulation of internal stochasticity by tuning the rates of transcription and translation can be utilized to cause the redistribution and to lead cells to the desired state in bimodal distribution^30^. This finding provided a new strategy to control the phenotypic variation through engineered gene circuits. In this work, we aim to examine how the change of the stochasticity, caused by the inhibitor, affects the bimodal distribution.

## Models

### The gene regulatory network and the deterministic model

**Figure 1:**
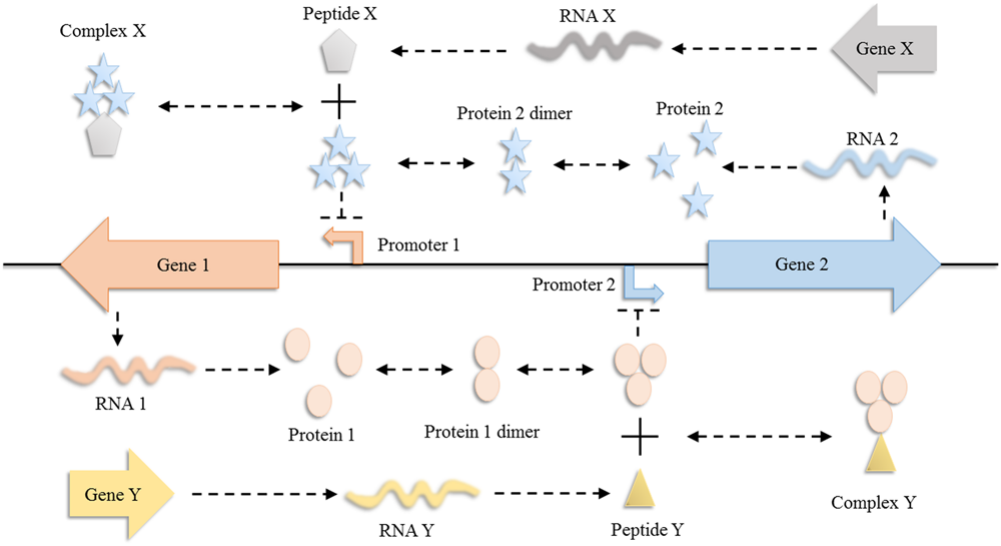
Reaction networks of the systems with bistability. (**a**) The mutually inhibitory network is one of the canonical motifs of genetic toggle switches, where one regulatory protein, P1 or P2, represses the expression of the gene encoding the other regulatory protein, P2 or P1. The subtle balance of P1 and P2 decides the binary fates. A cell stays at the state with either high expression of P1 or P2. There are two inhibitors, peptides X and Y, are ligands to P2 and P1. The binding of the inhibitor removes the repression of regulatory proteins. In other words, peptides X favors the expression of gene 1.

The genetic toggle switches are composed of two genes encoding regulatory proteins P1 and P2 which inhibit the expression of gene 2 and gene 1, respectively (Fig. 1). More precisely, the trimer of protein P2 represses gene 1, and the trimer of P1 represses gene 2. Such a reaction network, including two opposing fate-determining proteins, is one of the canonical motifs of bistable switches. This system also accounts for two inhibitors, the peptide Y acted on trimer of protein 1 and the peptide X on trimer of protein 2. Accordingly, the reactions of the system are listed in Supplementary Table S1. Following the mass action law, the deterministic model (Supplementary Table S2) was formulated in agreement with the reactions. The nomenclature and the values of parameters, which are based on literature^18, 22, 30–37^, are listed in Supplementary Tables S3 and S4, respectively. The steady state responses were obtained by functions **fsolve** or **solve** of the symbolic toolbox in Matlab.

### The Stochastic Model

Based on the reaction network shown in Fig. 1, we formulated the Chemical Master Equation (CME). Following the Stochastic Simulation Algorithm (SSA)^38^, we conducted the numerical analysis with the software SynBioSS^39^. Each simulation is composed of ten thousand trajectories (trials) and each trajectory is obtained by calculating the system to ninety thousand seconds. It was sampled only at the end point. The parameters of nominal values are listed in Supplementary Table S4. The cellular volume is of 10^−15^ liter. The initial conditions of proteins are given as the values of the unstable steady-state obtained from the deterministic model and the DNA starts with the active configuration. For other variables, they are zeros at beginning. Note that the initial conditions have no impact on final results as the system reached the stationary distribution. The outcome was processed by separating cells into two modes, the ON and the OFF. Cells with the level of protein monomer higher than that of the unstable steady state were considered as ON state, otherwise OFF state. The number of cells in each model was then counted. The “free energy” was calculated with accordance to literature^40^. Specifically, the “free energy” = −log (number of trajectories of (P1 − P2)).

## Results

The mutually inhibitory system, shown in Fig. 1, was examined. The reaction network is composed of two genes; each promoter is repressed by the protein produced from the other gene. The trimer of the regulatory protein P1 is the repressor on the promoter of gene 2. Similarly, P2 trimer represses gene 1. Consequently, the ON state of P1 leads to the OFF state of P2 and vice versa. This mutually inhibitory network is one of the canonical patterns of bistability^30^ and it performs bimodal distribution in population level as well.

Peptides Y and X are inhibitors to P1 and P2 trimers, respectively. The repression of P2 trimer on promoter 1 is lifted when the gene encoding peptide X is overexpressed. Consequently, the cell stays at the ON state of P1. The conventional beliefs suggest this outcome resulted from merely one factor, the consumption of P2 trimer by inhibitor X. However, in this work, we aim to unveil the role of the stochasticity in cell fate determination. More precisely, we explored how inhibitor exploits stochasticity to drive the cell to a specific state.

### The Bimodal Distribution of the System

**Figure 2:**
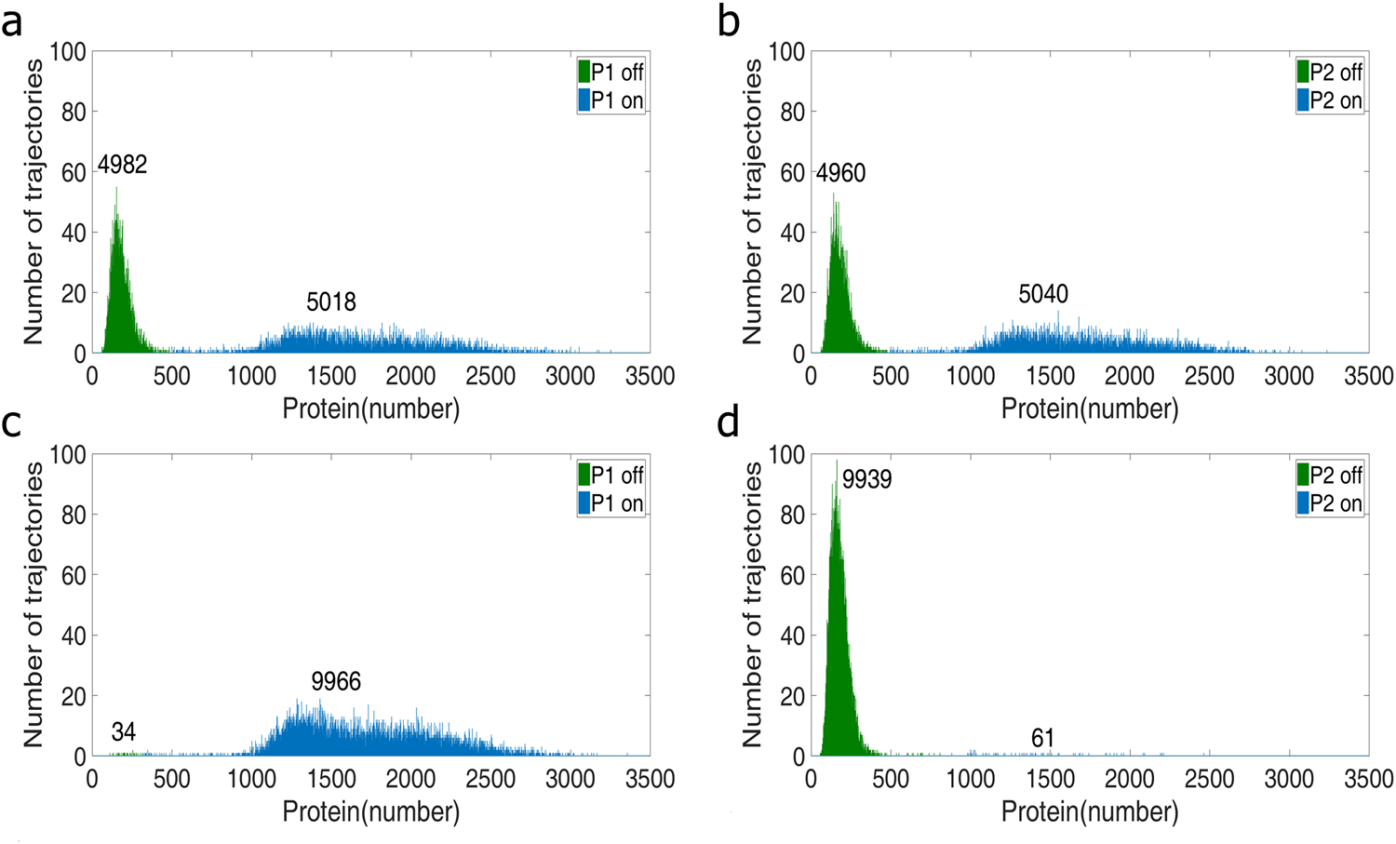
The bimodal distribution (**a**) and (**b**) are the stationary bimodal distribution of P1 and P2 monomers, obtained from parameters of nominal values. The number above each mode indicates the cell counts of the mode. The ON state is in blue color and OFF in green color. (**c**) and (**d**) are bimodal distributions with the transcription rate of gene X adjusted to 8 folds of the nominal value but that of gene Y is kept at the nominal value. The high level of the peptide X makes cells move to the ON state of P1 and OFF state of P2.

The bimodal distribution in population level was explored by the stochastic simulations. The stationary distributions were shown in Fig. 2a and b. The ON is in blue color and the OFF in green color; the number above the mode indicates the cell counts in the mode. As one of the features of the mutually inhibitory system, the number of cells at the ON state of P1 should nearly the same as that of the OFF state of P2. Moreover, in this study, the values of parameters for gene 1 is the same as that of gene 2 so the number of cells at the ON state of P1 should theoretically equal to that at ON state of P2. Therefore, for both P1 and P2, the number of cells at ON state is almost the same as that of cells at OFF state with parameters at nominal values.

The overexpression of gene X led to the high level of peptide X. Specifically, the transcription rate of gene X was adjusted to 8 folds of the nominal value and the distributions of P1 and P2 were shown in Fig. 2c and d, respectively. It is clear that the increase of peptide X, as an inhibitor to P2 trimer, pushing cells to the ON state of P1. More than 99% of cells were at P1 ON state or P2 OFF state.

### The Change of the Stochasticity by the Increase of Inhibitor Pushing Cells to the Specific State

**Figure 3:**
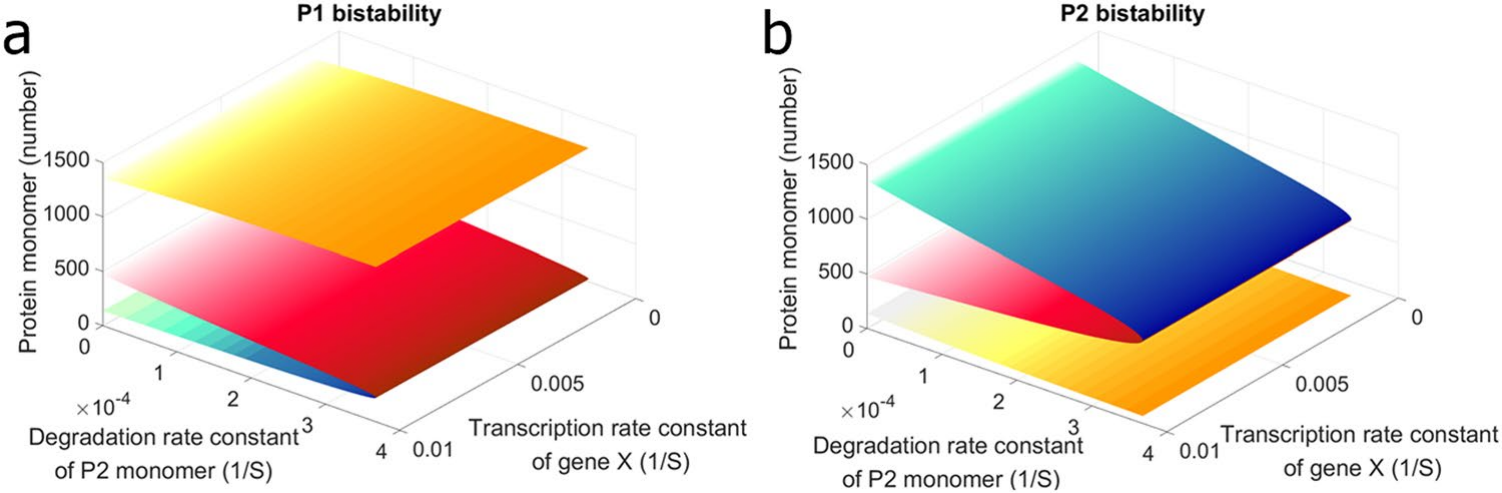
The bistable behaviors with the assumption that the only consumption of complexes X and Y comes from dissociation. The axis of the transcription rate indicates the expression level of gene X. Note that the shift along this axis caused no difference from the aspect of deterministic steady state analysis. The axis of the degradation rate represents the consumption rate of protein P2 monomer by degeneration. The movement along this axis altered the deterministic bistable behaviors.

**Figure 4:**
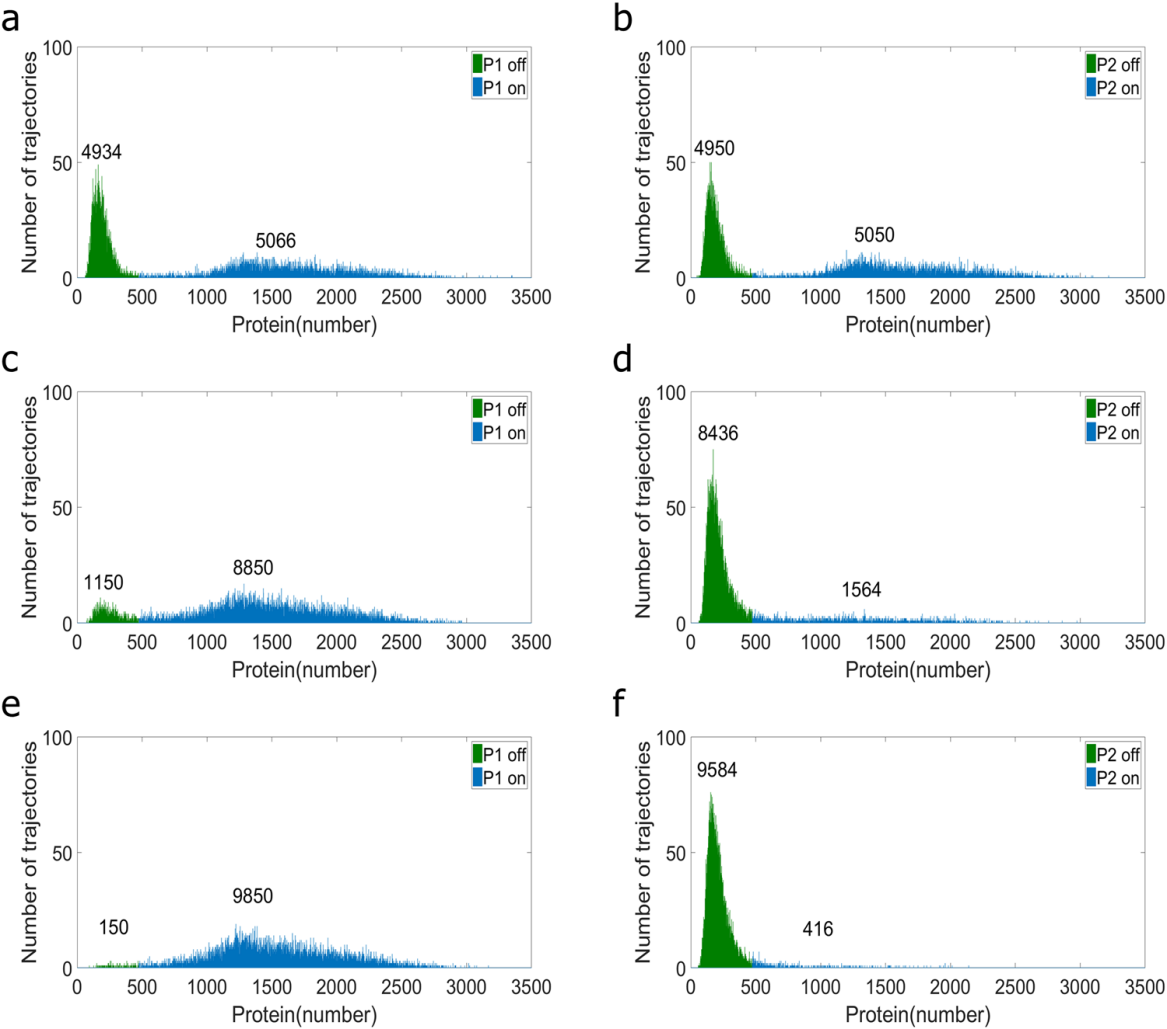
The bimodal distribution under the circumstance with the assumption that the only consumption of complexes X and Y comes from dissociation. There is no shift of the deterministic steady state and the change of stationary distribution results from altering the stochasticity. The transcription rate of gene Y is of the nominal value. (**a**) and (**b**) are bimodal distributions of P1 and P2 monomers with the transcription rate of gene X of the nominal value. (**c**) and (**d**) are with the transcription rate of gene X adjusted to 8 folds of the nominal value. Peptide X instructed cells to the ON state of P1 through the change of stochasticity. (**e**) and (**f**) are bimodal distributions with the transcription rate of gene X adjusted to 16 folds of the nominal value. Most of the cells were guided to the ON state of P1 by the change of intracellular stochasticity.

In addition to altering deterministic steady states, the inhibitor also caused the change of intracellular stochasticity. By using the term “change of stochasticity”, it means all kinds of changes captured by the stochastic model. To investigate how inhibitor exploited the stochasticity to drive cells to the specific state is of interest. It is, of course, not easy in practice to examine the influence of stochastic change on bimodal distribution alone but we can always take advantage of a mathematical model to achieve a better understanding. To this end, we assume that the only consumption of complexes X and Y comes from dissociation; as long as this assumption holds, from the deterministic steady state analysis, the level of free P2 trimer stays the same regardless the level of peptide X (Text S1). Figure 3 is the plot of bistability from steady state analysis of equations in Table S2. The surface in Fig. 3a and b represents the steady states of free P1 and P2 monomer, respectively. The red color indicates the unstable steady state. Along the axis of the transcription rate, which is the expression level of gene X, the bistable behaviors show no shifts. It implies that the deterministic approaches cannot capture the change of the stochasticity. Nevertheless, while applying stochastic simulations, the cells in bimodal distribution migrated to the ON state of P1. Figure 4a and b are the distributions of P1 and P2 with the transcription rate of gene X at nominal value. Remarkably, when the transcription rate of gene X increased to 8 folds of the nominal value (Fig. 4c and d), cells migrated to the ON state of P1 though there is no difference from the aspect of deterministic steady state analysis. Remarkably, it is also possible to guide nearly all cells to the specific state by intracellular stochasticity alone. As shown in Fig. 4e and f, about 99% of cells were driven to the ON state of P1 with the transcription rate of gene X as 16 folds of the nominal value.

### The Contribution of Intracellular Stochasticity of Guiding Cells to the Specific State

In nature, the inhibitor uses two ways to push cells to the specific state. One is through the consumption of P2 trimer and the other is by changing the intracellular stochasticity (Fig. 4c and d). The former altered the deterministic results but the later was only observed by stochastic simulations. Intriguingly, the latter appears to be influential in redistributing bimodal distribution.

**Figure 5:**
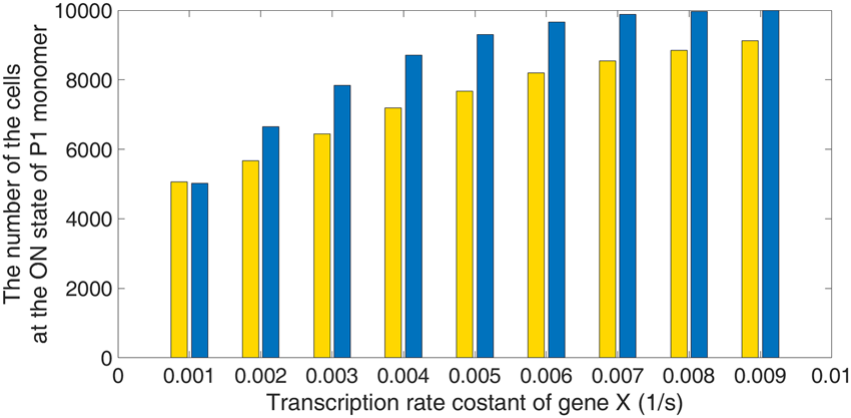
The influence of inhibitor on bimodal distribution. The y-axis is the number of cells at the ON state of P1 monomer. We examined the influence of inhibitor on bimodal distribution for two scenarios. For both scenarios, the transcription rate constant of Y is of nominal value and that of X is specified in the x-axis where 0.001 is the nominal value. The yellow bar indicates the circumstance with the assumption that the only consumption of complexes X and Y comes from dissociation. Namely, the yellow bar tells us how the change of stochasticity alone affects the bimodal distribution. In comparison to the blue bar which accounts for both the change of stochasticity and the consumption of P2 trimer, it concludes that the change of the stochasticity is also the main cause of pushing cells to the specific state.

The number of cells at the ON state of P1 for different levels of peptide X was plotted in Fig. 5. The yellow bar indicates the circumstance that the only consumption of complexes X and Y comes from dissociation; the blue bar represents the common situation as described by equations in Table S2. In other words, the yellow bar provides us an opportunity to observe how distributions were manipulated merely due to the change of stochasticity and the blue bar takes both the consumption of P2 trimer and the change of the stochasticity into consideration. For gene X at the transcription rate constant of 0.001 (1/s) which is the nominal value, cells are equally distributed into ON and OFF mode of P1. When the transcription rate constant of gene X increased, cells were pushed to the ON state of P1 for both scenarios. Remarkably, for the case accounts only for the change of the stochasticity, there are still a lot of cells at the ON state of P1. This outcome implies that the change of the stochasticity is also the main cause of driving cells to the desired state.

## Discussion

The inhibitor plays a crucial role in gene regulatory network but the mechanisms of how it affects the bimodal distribution were not well studied. We examined the influence of altering stochasticity on the bimodal distribution and found that it is also the main cause of driving cells to the desired state, as shown in Fig. 5. Such a change of stochasticity is moving along the axis of the transcription rate of gene X in Fig. 3, which shows no difference from the aspect of deterministic steady state analysis. In the other hand, the consumption of regulatory protein is moving along the axis of the degradation rate of P2 monomer, which alters the deterministic bistable behaviors. In a word, inhibitors successfully instructed cells to the specific state at least through these two movements.

The discovery of this study is not limited to the inhibitor. It unveiled the possibility of exploiting the change of stochasticity to control the state of cells in a bimodal distribution. The bimodal distribution raised from bistable switches exists in numerous biological systems^3–5, 14, 17, 18^ and how to effectively control the portion of cells in each mode is of high value. Unlike the conventional ways which deal with the bimodal distribution only by the deterministic approaches, our finding, from a new angle, suggested the role of stochasticity is critical. We demonstrated that the altering of stochasticity without consuming regulatory protein is sufficient to push nearly all cells to the specific state (Fig. 4c). It implied that the change of stochasticity, by the inhibitor, can be utilized as a method to redistribute cells. In our previous work^30^ we reported that the change of stochasticity, through manipulating the rate of transcription and translation, drove cells to the specific state. However, such an artificial method caused huge noise and compromised the bimodal distribution. The strategy reported in the presented work, from observing the natural interaction of inhibitors and regulatory proteins, does not suffer aforementioned drawbacks and is much easier to be applied. Note that the interaction between proteins and ligands is ubiquitous in biological systems and the proposed method is ready to be applied to all these. Moreover, the change of stochasticity is from the competition of reactions, which is everywhere in regulatory networks. One famous example of practicing the change of stochasticity on redistributing cells to the specific state in bimodal distribution is the novel approach of detecting the latent HIV^41^. The quiescent state is a big problem of detecting HIV infection, and the state switches by the change of the stochasticity offer a better solution. It is always beneficial to know how stochastic fluctuations influences the biological system.

**Figure 6:**
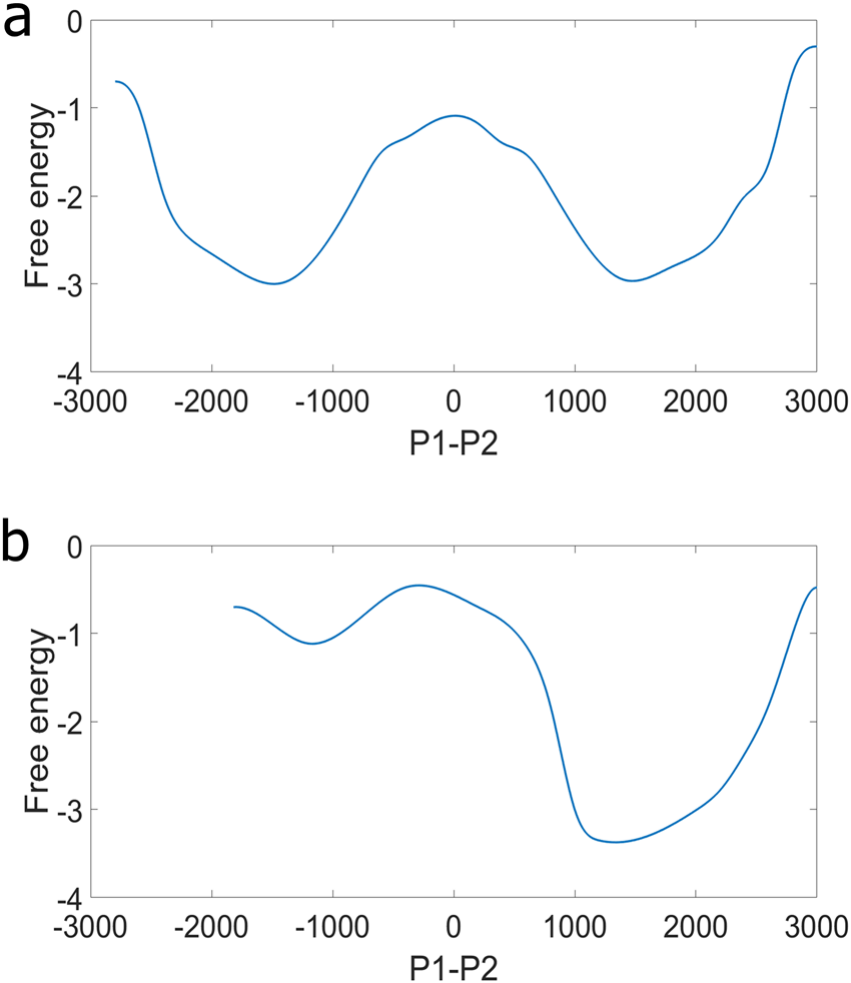
The “free energy” altered by the change of stochasticity. The x-axis represents the difference between P1 monomer and P2 monomer. For both sub-figures, the transcription rate constant of Y is of nominal value. (**a**) the transcription rate of gene X is of nominal value. (**b**) the transcription rate of gene X is 8 folds of the nominal value. The addition of inhibitor X lower the “free energy” of the right valley and raised that of the left valley; cells moved from left to right (which is from P1 OFF to P1 ON).

Instead of the protein distribution, “free energy”^40^ provides comprehension from a different angle. With the assumption that the only consumption of complexes X and Y comes from dissociation, we observed how the system was altered merely due to the change of stochasticity. Figure 6a is the “free energy” for the case which the transcription rate of gene X is of nominal value and Fig. 6b the case that the transcription rate of gene X is 8 folds of the nominal value. The left valley indicates the “free energy” of P1 OFF and the right valley is that of P1 ON. The ordinate zero of the y-axis is arbitrary but consistent within two sub-figures. The onset of the change of stochasticity by the addition of inhibitor lowered the “free energy” of the right valley (Fig. 6b) and cells incline to stay at the ON state of P1. Moreover, it also raised the “free energy” of the left valley and push cells away from P1 OFF state.

### Data availability statement

The model and parameters leading to the findings of this study are available in the manuscript and the supplementary information.

## Electronic supplementary material

Supplementary Information

## References

[1] Hasty J, McMillen D, Isaacs F, Collins JJ (2001). Computational studies of gene regulatory networks: in numero molecular biology. Nature Reviews Genetics.

[2] Kærn M, Elston TC, Blake WJ, Collins JJ (2005). Stochasticity in gene expression: from theories to phenotypes. Nature Reviews Genetics.

[3] Ferrell JE (2002). Self-perpetuating states in signal transduction: positive feedback, double-negative feedback and bistability. Current Opinion in Cell Biology.

[4] Gardner TS, Cantor CR, Collins JJ (2000). Construction of a genetic toggle switch in Escherichia coli. Nature.

[5] Ozbudak EM, Thattai M, Lim HN, Shraiman BI, van Oudenaarden A (2004). Multistability in the lactose utilization network of Escherichia coli. Nature.

[6] Ptashne, M. & Switch, A. G. P. Lambda and Higher Organisms. *Cell and Blackwell Scientific, Cambridge, MA* (1992).

[7] Zhang P (1999). Negative cross-talk between hematopoietic regulators: GATA proteins repress PU. 1. Proceedings of the National Academy of Sciences.

[8] Reddy VA (2002). Granulocyte inducer C/EBPα inactivates the myeloid master regulator PU. 1: possible role in lineage commitment decisions. Blood.

[9] Huang S, Guo Y-P, May G, Enver T (2007). Bifurcation dynamics in lineage-commitment in bipotent progenitor cells. Developmental biology.

[10] Huang S (2009). Reprogramming cell fates: reconciling rarity with robustness. Bioessays.

[11] Iwasaki H (2005). Distinctive and indispensable roles of PU. 1 in maintenance of hematopoietic stem cells and their differentiation. Blood.

[12] Chickarmane V, Peterson C (2008). A computational model for understanding stem cell, trophectoderm and endoderm lineage determination. PLoS one.

[13] Andrecut M, Halley JD, Winkler DA, Huang S (2011). A general model for binary cell fate decision gene circuits with degeneracy: indeterminacy and switch behavior in the absence of cooperativity. PloS one.

[14] Kepler TB, Elston TC (2001). Stochasticity in transcriptional regulation: Origins, consequences, and mathematical representations. Biophysical Journal.

[15] Maamar H, Dubnau D (2005). Bistability in the Bacillus subtilis K-state (competence) system requires a positive feedback loop. Molecular Microbiology.

[16] Yildirim N, Mackey MC (2003). Feedback regulation in the lactose operon: A mathematical modeling study and comparison with experimental data. Biophysical Journal.

[17] Kobayashi H (2004). Programmable cells: Interfacing natural and engineered gene networks. Proceedings of the National Academy of Sciences of the United States of America.

[18] Tian TH, Burrage K (2006). Stochastic models for regulatory networks of the genetic toggle switch. Proceedings of the National Academy of Sciences of the United States of America.

[19] Singh A, Weinberger LS (2009). Stochastic gene expression as a molecular switch for viral latency. Current opinion in microbiology.

[20] Song H-S, Ramkrishna D (2013). Complex nonlinear behavior in metabolic processes: Global bifurcation analysis of Escherichia coli growth on multiple substrates. Processes.

[21] Chatterjee A (2013). Antagonistic self-sensing and mate-sensing signaling controls antibiotic-resistance transfer. Proceedings of the National Academy of Sciences.

[22] Chatterjee A (2011). Convergent transcription confers a bistable switch in Enterococcus faecalis conjugation. Proceedings of the National Academy of Sciences.

[23] Shu C-C, Chatterjee A, Hu W-S, Ramkrishna D (2012). Modeling of gene regulatory processes by population-mediated signaling: New applications of population balances. Chemical engineering science.

[24] Shu C-C, Chatterjee A, Hu W-S, Ramkrishna D (2013). Role of Intracellular Stochasticity in Biofilm Growth. Insights from Population Balance Modeling. PloS one.

[25] Zhu, Z., Zheng, T., Lee, C. G., Homer, R. J. & Elias, J. A. in *Seminars in cell & developmental biology*. 121–128 (Elsevier). 10.1016/s1084-9521(02)00018-6 12127145

[26] Firman T, Ghosh K (2013). Competition enhances stochasticity in biochemical reactions. The Journal of chemical physics.

[27] Eldar A, Elowitz MB (2010). Functional roles for noise in genetic circuits. Nature.

[28] Xu Y, Zhu Y-n, Shen J, Su J (2014). Switch dynamics for stochastic model of genetic toggle switch. Physica A: Statistical Mechanics and its Applications.

[29] Xu, Y., Li, Y., Zhang, H., Li, X. & Kurths, J. The Switch in a Genetic Toggle System with Lévy Noise. *Scientific Reports* **6** (2016). 10.1038/srep31505 PMC4990962 27539010

[30] Shu C-C, Yeh C-C, Jhang W-S, Lo S-C (2016). Driving Cells to the Desired State in a Bimodal Distribution through Manipulation of Internal Noise with Biologically Practicable Approaches. PloS one.

[31] Henriksson-Peltola P, Sehlen W, Haggård-Ljungquist E (2007). Determination of the DNA-binding kinetics of three related but heteroimmune bacteriophage repressors using EMSA and SPR analysis. Nucleic acids research.

[32] Mehra S, Charaniya S, Takano E, Hu W-S (2008). A bistable gene switch for antibiotic biosynthesis: the butyrolactone regulon in Streptomyces coelicolor. PLoS One.

[33] Divita G, Rittinger K, Geourjon C, Deléage G, Goody RS (1995). Dimerization kinetics of HIV-1 and HIV-2 reverse transcriptase: a two step process. Journal of molecular biology.

[34] Ingr M, Uhlíková Tá, Stříšovský K, Majerová E, Konvalinka J (2003). Kinetics of the dimerization of retroviral proteases: the “fireman’s grip” and dimerization. Protein science.

[35] Markgren P-O (2001). Determination of interaction kinetic constants for HIV-1 protease inhibitors using optical biosensor technology. Analytical biochemistry.

[36] Shu C-C, Chatterjee A, Dunny G, Hu W-S, Ramkrishna D (2011). Bistability versus bimodal distributions in gene regulatory processes from population balance. PLoS Comput Biol.

[37] Sotiropoulos V, Kaznessis YN (2007). Synthetic tetracycline-inducible regulatory networks: computer-aided design of dynamic phenotypes. BMC Systems Biology.

[38] Gillespie DT (1977). Exact stochastic simulation of coupled chemical reactions. The journal of physical chemistry.

[39] Hill AD, Tomshine JR, Weeding EM, Sotiropoulos V, Kaznessis YN (2008). SynBioSS: the synthetic biology modeling suite. Bioinformatics.

[40] Warren PB, Ten Wolde PR (2004). Enhancement of the stability of genetic switches by overlapping upstream regulatory domains. Physical review letters.

[41] Dar RD, Hosmane NN, Arkin MR, Siliciano RF, Weinberger LS (2014). Screening for noise in gene expression identifies drug synergies. Science.

