# Supplementary material for: Inhibitors Alter the Stochasticity of Regulatory Proteins to Force Cells to Switch to the Other State in the Bistable System
Source: Sci Rep. 2017 Jun 30;7:4413. doi: 10.1038/s41598-017-04596-7 (PMC5493615; doi:10.1038/s41598-017-04596-7)
Supplement: Supplementary file 1 — Supplementary Information [file 41598_2017_4596_MOESM1_ESM.doc]

Supplementary Material

**Inhibitors Alter the Stochasticity of Regulatory Proteins to Force Cells to Switch to the Other State in the Bistable System**

Wun-Sin Jhang^&^, Shih-Chiang Lo^&^, Chen-Chao Yeh, Che-Chi Shu*

**Table S1: The reactions of the system**

| **(1)** |
| --- |
| **(2)** |
| **(3)** |
| **(4)** |
| **(5)** |
| **(6)** |
| **(7)** |
| **(8)** |
| **(9)** |
| **(10)** |
| **(11)** |
| **(12)** |
| **(13)** |
| **(14)** |
| **(15)** |
| **(16)** |
| **(17)** |
| **(18)** |

For Table S1, the reactions 1^st^ and 2^nd^ describe the transcription of the active and inactive conformation of gene 1, respectively. The reaction 3^rd^ indicates the translation of P1. The reactions 4^th^ and 5^th^ depict the process of protein monomer to dimer, dimer to trimer, respectively. The subscript 2 and 3 indicates the dimer and the trimer. The reaction 6^th^ describes the configuration change of gene 2 from active to inactive due to the binding of P1 trimer. The reactions 7^th^ and 8^th^ are the production of inhibitor Py, the transcription and translation processes, respectively. The interaction of inhibitor Py to the trimer of P1 is described by reaction 9^th^ where Y is the complex formed by Py and P1 timer. The reactions 10^th^ to 18^th^ are for gene 2. They include the expression of gene 2, the repression of gene 1 by P2 trimer and the complex X formed by Px and P2 trimer.

**Table S2: The mass-action equations of the system**

| $\frac{d[DA1]}{dt}=k_{-DI1}[DI1]-k_{DI1}[P2_{3}][DA1]$ **(1)** |
| --- |
| $\frac{d[DI1]}{dt}=k_{DI1}[P2_{3}][DA1]-k_{-DI1}[DI1]$ **(2)** |
| $\frac{d[R1]}{dt}=k_{RA1}[DA1]+k_{RI1}[DI1]-(k_{dR1}+\mu)[R1]$ **(3)** |
| **(4)** |
| $\frac{d[P1_{2}]}{dt}=k_{P1_{2}}[P1]^{2}-k_{-P1_{2}}[P1_{2}]-k_{P1_{3}}[P1_{2}][P1]+k_{-P1_{3}}[P1_{3}]-(k_{dP1_{2}}+\mu)[P1_{2}]$ **(5)** |
| $\frac{d[P1_{3}]}{dt}=k_{P1_{3}}[P1_{2}][P1]-k_{-P1_{3}}[P1_{3}]-k_{DI2}[P1_{3}][DA2]+k_{-DI2}[DI2]-k_{Y}[P1_{3}][Py]+k_{-Y}[Y]-(k_{dP1_{3}}+\mu)[P1_{3}]$ **(6)** |
| $\frac{d[Ry]}{dt}=k_{Ry}[DNAy]-(k_{dRy}+\mu)[Ry]$ **(7)** |
| $\frac{d[Py]}{dt}=k_{Py}[Ry]-k_{Y}[P1_{3}][Py]+k_{-Y}[Y]-(k_{dPy}+\mu)[Py]$ **(8)** |
| $\frac{d[Y]}{dt}=k_{Y}[P1_{3}][Py]-k_{-Y}[Y]-(k_{dY}+\mu)[Y]$ **(9)** |
| $\frac{d[DA2]}{dt}=k_{-DI2}[DI2]-k_{DI2}[P1_{3}][DA2]$ **(10)** |
| $\frac{d[DI2]}{dt}=k_{DI_{2}}[P1_{3}][DA2]-k_{-DI2}[DI2]$ **(11)** |
| $\frac{d[R2]}{dt}=k_{RA2}[DA2]+k_{RI2}[DI2]-(k_{dR2}+\mu)[R2]$ **(12)** |
| $\frac{d[P2]}{dt}=k_{P2}[R2]-2k_{P2_{2}}[P2]^{2}+2k_{-P2_{2}}[P2_{2}]-k_{P2_{3}}[P2_{2}][P2]+k_{-P2_{3}}[P2_{3}]-(k_{dP2}+\mu+k_{r})[P2]$ **(13)** |
| $\frac{d[P2_{2}]}{dt}=k_{P2_{2}}[P2]^{2}-k_{-P2_{2}}[P2_{2}]-k_{P2_{3}}[P2_{2}][P2]+k_{-P2_{3}}[P2_{3}]-(k_{dP2_{2}}+\mu)[P2_{2}]$ **(14)** |
| $\frac{d[P2_{3}]}{dt}=k_{P2_{3}}[P2_{2}][P2]-k_{-P2_{3}}[P2_{3}]-k_{DI1}[P2_{3}][\text{D}A1]+k_{-DI1}[DI1]-k_{X}[P2_{3}][Px]+k_{-X}[X]-(k_{dP2_{3}}+\mu)[P2_{3}]$ **(15)** |
| $\frac{d[Rx]}{dt}=k_{Rx}[DNAx]-(k_{dRx}+\mu)[Rx]$ **(16)** |
| $\frac{d[Px]}{dt}=k_{Px}[Rx]-k_{X}[P2_{3}][Px]+k_{-X}[X]-(k_{dPx}+\mu)[Px]$ **(17)** |
| $\frac{d[X]}{dt}=k_{X}[P2_{3}][Px]-k_{-X}[X]-(k_{dX}+\mu)[X]$ **(18)** |

For Table S2, the 1^st^ equation describes the DNA in an active conformation and the 2^nd^ equation indicates an inactive conformation. The equation 3^rd^ depicts the RNA of gene 1. The equations 4^th^ to 6^th^ are the changes of P1 monomer, dimer, and trimer. Note that the influence of Py on the trimer of P1 is included in equation 6^th^ as fifth and sixth terms. The equations 7^th^ and 8^th^ describe the formation of RNA of gene Y as well as the inhibitor Py, respectively. The equation 9^th^ describes the behavior of complex Y composed of P1 trimer and inhibitor Py. The equations 10^th^ to 18^th^ are similar to equations 1^st^ to 9^th^ but written for gene 2 and inhibitor Px.

**Table S**3: Nomenclature of the variables

| Annotation | Description |
| --- | --- |
| *DA_1_* | DNA of gene 1 in active formation |
| *DI_1_* | DNA of gene 1 in inactive formation |
| *R_1_* | RNA from gene 1 |
| *P1* | Protein encoded in gene 1 |
| *P1_2_* | P_1_ dimer |
| *P1_3_* | P_1_ trimer |
| *DNAy* | Gene y |
| *Ry* | RNA from gene y |
| *Py* | Peptide Y which is the inhibitor to P1 trimer |
| *Y* | Complex Y |
| *DA_2_* | DNA of gene 2 in active formation |
| *DI_2_* | DNA of gene 2 in inactive formation |
| *R_2_* | RNA from gene 2 |
| *P2* | Protein encoded in gene 2 |
| *P2_2_* | P_2_ dimer |
| *P2_3_* | P_2_ trimer |
| *DNAx* | Gene x |
| *Rx* | RNA from gene x |
| *Px* | Peptide X which is the inhibitor to P2 trimer |
| *X* | Complex X |

**Table S4: The values of parameters**

| **Parameter** | **Description** | **Value** | **Units** | **Ref.** |
| --- | --- | --- | --- | --- |
| $k_{DI 1}$ | Binding rate constant of P_2_ trimer to promoter 1 | 10^6^ | M^-1^S^-1^ | [^31^](#_ENREF_31)*^3^ |
| $k_{-DI1}$ | Dissociation rate constant of P_2_ trimer and promoter 1 | 10^-3^ | S^-1^ | [^31^](#_ENREF_31)*^3^ |
| $k_{RA 1}$ | Transcription rate constant of active gene 1 | 4.2x10^-1^ | S^-1^ | [^32^](#_ENREF_32) |
| $k_{RI 1}$ | Transcription rate constant of inactive gene 1 | 2x10^-2^ | S^-1^ | *1 |
| $k_{P 1}$ | Translation rate constant of P_1_ | 2.32x10^-3^ | S^-1^ | [^30^](#_ENREF_30) |
| $k_{P1_{2}}$ | Rate constant of forming P_1_ dimer | 7.4x10^2^ | M^-1^S^-1^ |  |
| $k_{-P1_{2}}$ | Dissociation rate constant of P_1_ dimer | 7.4x10^-3^ | S^-1^ | *2 |
| $k_{P1_{ 3}}$ | Rate constant of forming P_1_ trimer | 7.4x10^2^ | M^-1^S^-1^ |  |
| $k_{-P1_{ 3}}$ | Dissociation rate constant of P_1_ trimer | 7.4x10^-3^ | S^-1^ | *2 |
| $k_{Y}$ | Binding rate constant of P_1_ trimer to P_y_ | 10^6^ | M^-1^S^-1^ | [^35^](#_ENREF_35)*^3^ |
| $k_{-Y}$ | Unbinding rate constant of P_1_ trimer from P_y_ | 10^-3^ | S^-1^ | [^35^](#_ENREF_35)*^3^ |
| $k_{Ry}$ | Transcription rate constant of *R_y_* from *DNAy* | 10^-3^ | S^-1^ | [^22^](#_ENREF_22)*^3^ |
| $k_{P_{y}}$ | Translation rate constant of P_y_ | 10^-2^ | S^-1^ | [^36^](#_ENREF_36) |
| $k_{DI2}$ | Binding rate constant of P_1_ trimer to promoter 2 | 10^6^ | M^-1^S^-1^ | [^31^](#_ENREF_31)*^3^ |
| $k_{-DI2}$ | Dissociation rate constant of P_1_ trimer and promoter 2 | 10^-3^ | S^-1^ | [^31^](#_ENREF_31)*^3^ |
| $k_{RA 2}$ | Transcription rate constant of active gene 2 | 4.2x 10^-1^ | S^-1^ | [^32^](#_ENREF_32) |
| $k_{RI 2}$ | Transcription rate constant of inactive gene 2 | 2 x 10^-2^ | S^-1^ | *1 |
| $k_{P2}$ | Translation rate constant of P_2_ | 2.32x10^-3^ | S^-1^ | [^30^](#_ENREF_30) |
| $k_{P2_{2}}$ | Rate constant of forming P_2_ dimer | 7.4x10^2^ | M^-1^S^-1^ |  |
| $k_{-P2_{2}}$ | Dissociation rate constant of P_2_ dimer | 7.4x10^-3^ | S^-1^ | *2 |
| $k_{P2_{3}}$ | Rate constant of forming P_2_ trimer | 7.4x10^2^ | M^-1^S^-1^ |  |
| $k_{-P2_{3}}$ | Dissociation rate constant of P_2_ trimer | 7.4x10^-3^ | S^-1^ | *2 |
| $k_{X}$ | Binding rate constant of P_2_ trimer to P_x_ | 10^6^ | M^-1^S^-1^ | [^35^](#_ENREF_35)*^3^ |
| $k_{-X}$ | Unbinding rate constant of P_2_ trimer from P_x_ | 10^-3^ | S^-1^ | [^35^](#_ENREF_35)*^3^ |
| $k_{Rx}$ | Transcription rate constant of *R_x_* from *DNAx* | 10^-3^ | S^-1^ | [^22^](#_ENREF_22)*^3^ |
| $k_{P_{x}}$ | Translation rate constant of P_x_ | 10^-2^ | S^-1^ | [^36^](#_ENREF_36) |
| $k_{dR 1}$ | Degradation rate constant of R_1_ | 6.15x10^-4^ | S^-1^ |  |
| $k_{dP 1}$ | Degradation rate constant of P_1_ | 1.63x10^-6^ | S^-1^ | *3 |
| $k_{dP1_{2}}$ | Degradation rate constant of P_1_ dimer | 10^-4^ | S^-1^ | [^37^](#_ENREF_37)*^3^ |
| $k_{dP1_{3}}$ | Degradation rate constant of P_1_ trimer | 10^-4^ | S^-1^ | [^37^](#_ENREF_37)*^3^ |
| $k_{dRy}$ | Degradation rate constant of *R_y_* | 6.15x10^-4^ | S^-1^ | [^30^](#_ENREF_30) |
| $k_{dP_{y}}$ | Degradation rate constant of P_y_ | 1.63x10^-6^ | S^-1^ | [^30^](#_ENREF_30) |
| $k_{dY}$ | Degradation rate constant of Y complex | 10^-4^ | S^-1^ | [^37^](#_ENREF_37)*^3^ |
| $k_{dR 2}$ | Degradation rate constant of R_2_ | 6.15x10^-4^ | S^-1^ |  |
| $k_{dP2}$ | Degradation rate constant of P_2_ | 1.63x10^-6^ | S^-1^ | *3 |
| $k_{dP2_{2}}$ | Degradation rate constant of P_2_ dimer | 10^-4^ | S^-1^ | [^37^](#_ENREF_37)*^3^ |
| $k_{dP2_{3}}$ | Degradation rate constant of P_2_ trimer | 10^-4^ | S^-1^ | [^37^](#_ENREF_37)*^3^ |
|  | Degradation rate constant of *R_x_* | 6.15x10^-4^ | S^-1^ | [^30^](#_ENREF_30) |
| $k_{dP_{x}}$ | Degradation rate constant of P_x_ | 1.63x10^-6^ | S^-1^ | [^30^](#_ENREF_30) |
| $k_{dX}$ | Degradation rate constant of X complex | 10^-4^ | S^-1^ | [^37^](#_ENREF_37)*^3^ |
| μ | Specific growth rate constant | 3.85x10^-4^ | S^-1^ | [^30^](#_ENREF_30) |

*^1^ We took the transcription rate constant of the active gene from [^32^](#_ENREF_32). In order to have the same ratio of active to inactive rate constant in [^30^](#_ENREF_30), the rate constant of the inactive gene is decided.

*^2^ We took the rate constant of the forward reaction from [^33^](#_ENREF_33). In order to keep the equilibrium rate constant the same as that in [^30^](#_ENREF_30), the rate constant of the reverse reaction is decided.

*^3^ Instead of taking the whole value, we took only the order of the parameter value.

**Text S1**

Complex X is involved in two equations, Eqs (15) and (18), in Table S2. With the assumption that the only consumption of complex X is the reaction of dissociation, the equations were rewritten as follows.

$\frac{d[P2_{3}]}{dt}=k_{P2_{3}}[P2_{2}][P2]-k_{-P2_{3}}[P2_{3}]-k_{DI1}[P2_{3}][\text{D}A1]+k_{-DI1}[DI1]-k_{X}[P2_{3}][Px]+k_{-X}[X]-(k_{dP2_{3}}+\mu)[P2_{3}]$ (S1)

$\frac{d[X]}{dt}=k_{X}[P2_{3}][Px]-k_{-X}[X]$ (S2)

If the system reaches steady state,$\frac{d[P2_{3}]}{dt}$ and $\frac{d[X]}{dt}$ are zero. Then the mass balance becomes:

$0=k_{P2_{3}}[P2_{2}][P2]-k_{-P2_{3}}[P2_{3}]-k_{DI1}[P2_{3}][\text{D}A1]+k_{-DI1}[DI1]-k_{X}[P2_{3}][Px]+k_{-X}[X]-(k_{dP2_{3}}+\mu)[P2_{3}]$ (S3)

$0=k_{X}[P2_{3}][Px]-k_{-X}[X]$ (S4)

Then we substituted Eq (S4) into Eq (S3) and got Eq (S5).

$0=k_{P2_{3}}[P2_{2}][P2]-k_{-P2_{3}}[P2_{3}]-k_{DI1}[P2_{3}][\text{D}A1]+k_{-DI1}[DI1]-(k_{dP2_{3}}+\mu)[P2_{3}]$ (S5)

Eq (S5) suggests that the concentration of complex X shows no effect on P2 trimer.
